# Explaining the concept of maternal health information verification and assessment during pregnancy: a qualitative study

**DOI:** 10.1186/s12884-021-03715-7

**Published:** 2021-03-26

**Authors:** Safoura Taheri, Mahmoud Tavousi, Zohre Momenimovahed, Ashraf Direkvand-Moghadam, Nazanin Rezaei, Nasibeh Sharifi, Ziba Taghizadeh

**Affiliations:** 1 Department of Midwifery, School of Nursing & Midwifery, Ilam University of Medical Sciences, Ilam, Iran; 2 Health Metrics Research Center, Iranian Institute for Health Sciences Research, ACECR, Tehran, Iran; 3 Department of Midwifery, School of Nursing and Midwifery, Qom University of Medical Sciences, Qom, Iran; 4 Department of Midwifery and Reproductive Health, School of Nursing and Midwifery, Tehran University of Medical Sciences, Tehran, Iran

**Keywords:** Health literacy, Verification and assessment of maternal health information, Pregnancy, Qualitative study, Iran

## Abstract

**Background:**

Pregnant women use information sources for their own health and health of their children. However, despite the importance of trusting the information sources, pregnant women may not have the ability to verify the maternal health information, which could have negative consequences for their health. The purpose of this study was to explain the concept of maternal health information verification and assessment in pregnant women according to their experiences and perception.

**Methods:**

This is a qualitative study that was conducted in 2017 in Tehran, Iran. The participants in this study consisted of 19 pregnant women who were selected by purposeful sampling. To collect data, semi-structured, in-depth and face to face interviews were conducted with participants and continued until saturation of data. Conventional content analysis method was used to analyze the data and to identify concepts and synthesize them into general classes. MAXQDA software version 10 was used to manage the data.

**Results:**

In the process of data analysis, the concept of verification and assessment of maternal health information in pregnancy was explained in two main categories, including “Validity of information resources” and “Reliance on information resources.” The category of Validity of information resources had two subcategories of valid and invalid sources, and the main category of Reliance on information resources had two subcategories of indicators of assurance, and confusion and trying to obtain assurance.

**Conclusion:**

The results indicated that pregnant women used various sources and indicators, as well as different evaluation methods to obtain information and verify it, especially when they are confused. Thus, health authorities and healthcare professionals should provide appropriate programs to familiarize mothers with credible sources, train pregnant women on standards and practices for judging the accuracy of information, and create a safe margin of information.

## Background

Pregnancy is a transforming time in every woman’s life. During this time, woman’s body changes, and she began to have many questions about the infant and the new lifestyle that awaits the family. Most women want assurance about their pregnancy being normal and thus, seek information in order to feel a sense of security [1]. According to the evidence, pregnant women use different sources of information on their own health and health of their children [2]. The information obtained from social networks affects the pregnancy-related health behaviors [3, 4], and pregnant women follow recommendations presented in social networks for prenatal care than by health care providers [3]. A study showed that over a period of 1 week, about 64%(64/ (100of pregnant women access the Internet by mobile phones and 82%(82/ 100) by computers [5]. Lima-Pereira [6] found that the Internet is the most common source of information during pregnancy after physicians.

One of the important factors in relation to maternal health information is trusting the resources and information provided by them [7]. In many studies, pregnant women have referred to Internet as a reliable source of information [8–12], while more than half of women in the study of Huberty et al. [13] referred online information as inaccurate or misleading. Studies have shown that a great deal of maternal health information on the Internet are not valid [14, 15], despite patient’s trusting them without any proof concerning their legitimacy [16, 17]. Today, access to maternal health information is easy and readily available at anytime and anywhere. Thus, it is important that individuals should be able to assess maternal health information related to their conditions [18]. It should be mentioned that consumers of maternal health information are aware that all sources of information do not necessarily have the same quality, but the challenge most people face is judging which information is more credible [19].

The ability of pregnant women in finding and understanding prenatal care information is critical to their health and health of their children; this need is especially important in women who may have a low level of health literacy [20]. Low-level health literacy is associated with limited ability to evaluate information using online search techniques [21, 22]. Evidence suggests that in the field of prenatal care, the majority of pregnant mothers do not have the ability to assess the credibility of information obtained from various websites [6, 23].

Most of the studies conducted on evaluation and assurance of maternal health information, have used quantitative approach, and no qualitative study has been done on this subject. Thus, given the importance of maternal health literacy for pregnant women, the aim of this study was to explain the concept of maternal health information verification and assessment in pregnant women through a qualitative study.

## Methods

A qualitative design was used based on a content analysis approach to reach the objectives of this study. Qualitative studies are used to improve our understanding of the world of human experience [24]. Content analysis is a systematic approach to categorization that can be used to explore a large amount of text information to identify trends and communication patterns [25]. The principles of explanatory description method [26] were used to answer the research question, which was: “What is the concept of maternal health information verification and assessment according to the experiences of pregnant women”? Qualitative research method is a guide for describing and interpreting how people deal with a phenomenon, especially in the field of health and treatment. The explanatory description provides a qualitative evolutionary approach to addressing clinical health phenomenon [26]. It provides a flexible structure for describing or understanding a phenomenon from the perspective of those who have experience in that area [26]. This description explains a number of factors including cultural, social, economic, political or evolutionary factors. After creating an explanatory description, nursing knowledge and practice can be effectively used [27].

This article is part of a PhD thesis in Sexuality and Reproductive Health. Ethical approval was obtained from Tehran University of Medical Sciences with the code: IR.TUMS.VCR.REC.1395.

### Study setting and participants

With a qualitative content analysis approach, this study was conducted between February and August 2017 to explain the experience of pregnant women in assessing the credibility of maternal health information in Tehran. Participants in this study consisted of 19 pregnant women who were selected by purposeful sampling. The participants were invited to the study when they came to receive pregnancy care or, they were introduced to us by midwives or other pregnant women. Individuals selected for the interviews had adequate experienced about the study topic and met the inclusion criteria (the desire to participate in the study, speaking Persian as their first language and being able to read and write). Sampling was done among pregnant women with various ages, education level, occupations and fertility records. It should be noted that, women with known psychological disorders and those with the experience of adverse life event (such as lost of a loved one, etc) in the last 6 months were not included in the study.

### Data collection

In this study, semi-structured, individual and in-depth interviews were developed to collect information. The participants were informed of the aim and method of study and then, were asked to provide an informed consent. The assurance was also given to them about confidentiality of their information and the right to withdraw from the study at any time. Nineteen semi-structured, individual and in-depth interviews were conducted with 19 pregnant women until data saturation. Interviews were conducted individually in a private room, and lasted for 45 to 90 min on average. The place of interview such as hospital or public health center was coordinated with the participants. The interview guide used in this study was developed for this study and has never been used before. The guiding questions were designed by the researchers according to the purpose of the study and the questions were developed during the interviews (Table 1). In this study, the interview guide was used that included questions on how to ensure the accuracy of health information during pregnancy. The main question was how did/do you know that the information you get from different sources is/was correct? The rest of the questions were designed based on the interview process and the responses of each individual. By listening to the interviews and reviewing the text of unstructured interviews, questions were raised in the interviewer’s minds for subsequent interviews. Probing questions such as: “What did you mean”? and “Can you explain more”? were also used to obtain more information, clarify the subject and encourage the participant to continue the interview. Interviews were audio recorded and written down on paper at the first opportunity. The interviews’ transcripts were checked against the audio files to ensure their accuracy. In the present study, to validate data, long-term engagement with the data was maintained and enough time was allocated to collect and analyze the data in order to collect richer data. Also, selecting participants with different experiences, aggregating an adequate amount of data and describing the formation of code, category and subcategory were used for data validity. The trustworthiness of this study was assured using Lincoln and Guba’s criteria [28]. To meet these criteria, activities such as purposeful sampling, member checking, peer checking, prolonged engagement with the subjects, and maintaining an audit trail by the corresponding author’s memos were carried out. The “member checking” was performed where the overall results of the study were checked again for each participant. Five experts in reproductive health and health literacy responded to the request for member checking.

**Table 1:** The questions guide used in this study

1	What did you want to know when you got pregnant? What information did you need? What information do you think a woman who gets pregnant should have? Need to have?
2	From whom did you get this information? From where? How did you determine that it is correct and appropriate? Please explain to me? Please tell me your experiences?
3	Search questions: Can you explain more about this? Can you give an example? For example, one of the topics you learned about, what steps did you take to ensure and apply what you learned? May you give an example of this? Please explain more to me.

### Data analysis

Hsieh and Shannon’s process of conventional content analysis was used to encode the data, identify concepts, and synthesize them into general classes [29]. From the three variations of content analysis, the conventional content analysis is identified as an analytical strategy that is appropriate for descriptive studies, where there are few theoretical models available to describe the phenomenon under study [29]. Conventional content analysis method was used to analyze the data through MAXQDA software version 10. Five-step analysis method of Graneheim and Lundman [30] was used to analyze the content of qualitative data. This method avoids the use of predefined category and allows the category and their names to be extracted from the data. At first, data analysis begins with frequent reading of the text to gain a general sense of understanding, and then it is read word-by-word to extract semantic units and synonyms are termed as codes. In the next stage, the codes are sorted into sub-categories taking into account their similarities and differences and finally, main categories as the expression of latent content of the text are formulated. The initial classification started from the second interview. Increased number of interviews also increased the number of codes, and then, by comparing and merging them, subcategories and main categories were formed. The researcher ensured that, the subcategories and categories were reviewed by the members of research team and the participants.

## Results

### Characteristics of the participants

The participants in this study consisted of 19 pregnant women with the average age of 29.8 years and range of 16–45 years. Among the pregnant women, 11 were nulliparous and 8 were multiparous. The average gestational age of women was 27 weeks with a range of 7–39 weeks. The highest education level of them was PhD student and the lowest was secondary school, and most of them were housewives. All participants were Iranian and Persian language was their first language. They also had the ability to read and write. The demographic characteristics of the participants are presented in Table 2. Abbreviations were used in the text to identify which contributors the quotes belonged to and also to keep the names of the individuals confidential.

**Table 2:** demographic characteristics of the participants

Code	Education	Employment status	Pregnancy number	Gestational age	Place of receiving care	Participation in delivery preparation classes
1	PhD student	Employed	First	29	–	No
2	MSc student	Employed	Third	20	Private GP clinic	No
3	Associate Degree	Housekeeper	First	24	Hospital	Yes
4	Diploma	Employed	Second	23	Hospital	No
5	BSc	Housekeeper	First	20	Hospital	No
6	Diploma	Housekeeper	Second	35	Hospital	No
7	third grade high school	Housekeeper	Third	37	Health center + hospital	No
8	Diploma	Housekeeper	Fourth	7	Health center	No
9	Diploma	Housekeeper	First	13	Health center + Private GP clinic	No
10	MSc	Employed	First	36	Hospital	Yes
11	third grade High school	Student	First	18	Health center	No
12	Third grade High school	Student	First	33	Health center	Yes
13	BSc	Housekeeper	Third	26	Health center + Private GP clinic	No
14	BSc	Employed	Second	38	Hospital + Health Center	Yes
15	BSc	Employed	Third	39	Hospital	No
16	Pre-university	Student	First	37	Hospital	Yes
17	Diploma	Housekeeper	First	24	Hospital + GP private clinic	No
18	BSc student	Employed	First	32	Hospital	Yes
19	BSc	Employed	First	22	GP private clinic	No

### Identified categories

Data analysis led to the emergence of two main categories, including “Validity of information resources” and “Reliance on information resources”. The main categories also had four subcategories (Table 3).

**Table 3:** The main categories and subcategories derived from the experience of mothers in the verification and assessment of maternal health information

Categories	Subcategories	Codes
Validity of information resources	Invalid sources	-Lack of trust in the internet resources given their being virtual and the existence of errors -Non-updated and incomplete information of the people around them for today’s world -Non-reference to some sources due to non-belief in their scientific basis -Lack of trust in the internet due to no-consideration of individual differences
	Valid source	-Book is a reliable source for mothers -Full trust in the elderly given the belief in their wisdom Considering those around them as equal to the doctor in scientific regard -The priority of receiving advice from health professionals compared to other sources -Belief in the correctness of all contents of health network -The importance of the experiences of individuals despite the lack of scientific proof
Reliance on information resources	Indicators of assurance	-Complete source, how to write a resource, Simple and understandable maternal health information, -Trust the scientific, professional and up-to-date source of information citing the source of the content -Generalization and similarity of the information received -The frequency of a content heard as a standard for the correctness of it -Accepting more proper information with individual circumstances -Experience, being welcomed and approval of others -Acceptance of information obtained based on reason and logic -Selecting a resource without a specific criterion
	Confusion and trying to obtain assurance	-Searching and recognizing valid sources to get information -Asking health professionals to confirm information from other sources Comparing the information received -Searching multiple sources to achieve consensus and confidence -Search for the validity of the source expressed in maternal health information -Ignore the very slight differences in the content of different sources

### Validity of information resources

The participants divided the information resources into two categories of valid sources and invalid sources.

### Invalid sources

Some participants considered the Internet and the recommendations of family members and friends as invalid sources. Internet websites were viewed by some mothers as invalid source of information due to reasons such as; inability of mothers to get answer for their questions, the probability of errors, non-scientific nature of some websites, and non-consideration of individual differences in the presentation of information. The recommendations of friends and relatives were also considered invalid by mothers for reasons, such as their information not being up-to-date.

P13: I do not have much confidence in the Internet because some Internet information is wrong. It is possible for the opposite to be true regarding the information you get, and it may not be the same for all women……

P4: Among the relatives, I do not accept what middle-aged women say because they speak like old people... I do not believe it.

### Valid source

According to the participants, valid sources used to get information were different, but despite the differences, healthcare professionals were a reliable source for them to obtain complete and safe information compared to other resources. It should be noted that even among healthcare professionals, sometimes information presented by them on the same topic were different.

P5: Look, there are things that you need to be sure of and you need experts. For example, if I go to a midwife and she says yes, this happens to you and it is natural…well, I accept it.

Participants pointed out the importance of paying attention to and using the opinions of informed people. They considered experience, knowledge and science of people around them as a reason to accept them equal to or more than healthcare professionals:

P17: I asked something and my mother said that there was no problem. It is not normal, so I did not ask anyone else. My mother answered me. I accept her, even as much as I accept my doctor.

Another interesting finding in this study was the women’s emphasize on the value of experiences of other people, even if there is no scientific proof of that experience. Participant number ten said: A series of information are experimental information…They have little scientific basis, but since they come from experience, they can still be used…

### Reliance on information resources

One of the concepts extracted from the experiences of participants regarding the verification and assessment of maternal health information was “the concept of Reliance on information resources”. Participants used criteria and methods to assess the credibility of information to ensure that they are valid.

### Indicators of assurance

Some participants had some criteria to ensure the information is valid. These criteria included: being from a complete source, how to write a resource, being simple and understandable, trust the scientific, professional and up-to-date sources of information, citing the source of content, generalization and similarity of information received, frequency of the content heard as a criterion for its correctness, accepting information with individual circumstances, coming from experience, being approved by others, and being reasonable and logical. Some of participants had no specific criteria and randomly received information from sources such as web sites. It may be said that, these mothers were the only ones who were not attentive to the assessment of information validity.

The completeness of information source was one of the indicators of pregnant women for assessing maternal health information.

P11: For example, I research about how sex is investigated in pregnancy or about fetal development ...There are good things written about them, but sometimes they are not perfect. You have to search a few places to find everything you want…

P10: TV and health network help a lot ... I was searching about pregnancy information and the quality of the programs, and I found that the health network has rich information compared to the television networks …

How to write, present and evaluate maternal health information in various sources, especially Internet resources was mentioned by the participants. The participants stated that, inaudible terms in websites designed by professionals, and even on sources such as television, are very few and they try to publish information in a manner that everyone can understand.

P12: The internet is a good source, and its content can be read and understood in any language and any age ….

P8: Doctors who talk about health and disease on television speak a simple and understandable language for all people.

The participants referred to the specialized and up-to-date source of information and believed that, the acceptance of information depends on the scientific nature of its resource, especially when there is confusion about different information.

P1: The radiologist who conducted ultrasound on me for NT had the license for it. It was very important to me ... I could not trust someone just because he/she is a sonologist... I tried to search for my answer rather than asking someone, because his/her information may not be up-to-date and may mislead me.

One of the standards that most participants expressed was to ensure the information they have gained is common and the same as information presented by other sources. This criterion, since it looks into resources, especially during confusion, gradually affects the ability of mothers to determine the accuracy of information.

P16: Well, when you search in many sources, naturally you get answer for your question.

Another finding of this study was that, in regard to contradictory information, some mothers preferred to select information that was more consistent with their current conditions.

P7: I have a sister who said; if you have sex when you approach the last month of your pregnancy, you will give birth more easily. She said she had heard it from our cousin. I read a book that opposed that idea…So I listened to what the book said, because I could not do it when I was 28 weeks pregnant as it was hard for me.

In a number of mothers, hearing information on a topic in different sources for several times could help them to ensure the accuracy of information.

P8: To check whether or not the information I received is correct, I check it by how many times I have heard it. If I have heard it many times, I think it is correct.

Moreover, the participants trusted the information sources if they have been acceptance by other mothers and mentioned by them.

P10: I try to read the books that others have read and were satisfied with them. I ask several people both from staff and from those who have had childbirth experience to make sure the information is valid... For example, internet sources are very visitor-friendly and I believe them to be more reliable.

Results showed that when judging the information, some mothers paid attention to the fact that whether the information is reasonable and logical or not, and then accepted or rejected them.

P10: In the end, the information I find after searching, I assess it with my logic. There were cases where I reached an answer but after assessing with my own logic, I came to the conclusion that it was not right.

One finding of this study was that in relation to conflicting information, some mothers preferred to select information sources that are more in line with their current situation and more applicable to them.

P7: My sister said that if I have more sex in last month of my pregnancy, I will have an easier delivery. I asked who had told her, she said she had heard it from friends. Then I read a book and realized that is not true... I tried to follow the book, because I couldn't have sex as I was in 7 months of pregnancy.

One mother stated that studying and confirming the events that occurred to her during pregnancy made her more confident in the source of information.

P10: I search the internet every week from the first week of pregnancy. For example, I had a burning feeling in my stomach this week, and in the internet I found that, if you have a burning sensation in the stomach this week, you should not be worried. This makes me to trust that website.

Another interesting finding was the emphasis the mothers had on the value of other people’s experiences, even if there was no scientific evidence for them. Participant 10 said that, there are series of information which are experiential...they are not so scientific, but they are still experience and can be used. Using everything, even small things, is good.

When choosing resources, some participants, especially in relation to various websites did not have a standard to select and receive information from a source and chose the information source randomly.

P7: When you search, there are a few websites that are not the same. I do not read all of them. I only read some that are different. I click at random to see what they say, and I do not read all of them.

Confusion and trying to obtain assurance participants had used some methods for verifying information to ensure the validity of maternal health information, especially during confusion. Based on the experiences of participants, one of the important measures to avoid confusion in information assessment is the recognition and selection of valid sources for maternal health information.

P1: I have not faced contradictory information, because I probably had access to good resources. In fact, I knew, for example, if I had a problem, then of course, the source could give me the right information.

One of the measures taken by most participants to ensure the accuracy of information they received was asking several sources the same question in order to reach a consensus and assurance:

P3: I first look at the Internet, and then I check other places for reassurance….

Another way of information assessment used by most participants was asking healthcare professionals to confirm information from other sources. On the other hand, it was argued that searching in non-physician resources was merely for getting extra information on the subject, and the physician was the main source for their decision-making.

P19: No matter how many places I search; I finally listen to my doctor. The reason I am doing searching is just to learn more about the subject.

Comparison of multi-source information was also a way of assessing the maternal health information obtained by the pregnant women

P12: Well, I compare some types of answers with each other; for example, doctor, midwife, and the Internet to see which one is better to choose...

A participant also argued that when facing contradicting information, she would ignore minor contradictions.

P5: I read two or three programs that had similar information about the size of fetus. For example, one of them stated, it should be 433 gr, another stated it should be 354 gr, so I ignored the difference.

## Discussion

The results of this study showed that pregnant women evaluated the validity of maternal health information by distinguishing the resources, as well as considering the indicators and practices to ensure the accuracy of information, especially during the confusion over various sources of information.

In the present study, some mothers considered the internet as invalid source of information for many reasons, such as the existence of errors, lack of knowledge, and not taking into account the individual differences between pregnant women. However, most of them had used the internet to obtain information and verify information obtained from other sources. Some studies have shown that most pregnant women see the internet as a very reliable source of information [12, 31, 32], while Huberty et al. [13] found that women were confused about using online information. Morahan-Martin [33] found that, the users usually seek for maternal health information only on online general search engines. Artieta-Pinedo [34] found that the websites of health care institutions or academic institutions contained the most reliable information. In our study, the majority of mothers had used public websites to search for information and did not know the medical websites with high credibility, and this increased their confusion over information.

Some participants referred to other sources such as books, papers, experiences of relatives, and TV health channels as valid sources. Some people considered information sources such as online associations and blogs as valuable sources for knowing the experiences of others. They also referred to healthcare professionals because of their medical knowledge, and books because of their scientific nature. Although these people had a different understanding of value, sources did not have a competitive role in their lives but complemented each other [2]. These findings, consistent with our study, show that mothers use different sources at first, but if needed; they look for source that confirms their point of view, which is often a healthcare professional. Regarding this, the results of Kassim [35] study suggested that in seeking information, the sensitivity of a subject for the individual is effective in determining how valid a source is.

Despite the priority of healthcare professionals, participants considered the recommendations and experiences of those around them as valid. In a study by Schölmerich et al. [36] all respondents emphasized that social network is an important source of support, especially during pregnancy and postpartum. All respondents introduced their mothers and sisters as the first reference for their questions at that time, and then healthcare professionals as the second reference when they are not satisfied with the information they received. Thus, designing programs to establish effective communication between healthcare professionals and social networks is important for promoting maternal health. The importance of these networks in the health of mothers should not be ignored as they can provide proper support platforms for pregnant women. In this study, the participants considered some indicators for creating a safe margin in maternal health information. In our study, generalization and similarity of information among different sources were the criteria that most participants had mentioned. Larsson [12] reported the source of information coherent with other sources and the source of information as the most important factors used by the majority of participants in evaluating the reliability of information. Moreover, the similarity in information in various websites increases the trust in individuals [35, 37]. However, it should be noted that the existence of some maternal health information on several websites may cause people to think they have verified an article by reading two sources. In this regard, some participants pointed out that some of the contents in some websites are replicated and copied, and that reduces their trust in them.

In this study, very few participants mentioned examining the cited source and updated information in their comments. Fox [38] found that 75% of people do not regularly review online maternal health information by using indexes such as the release date or the source of information. Another study showed that a few pregnant mothers examined the date of publication or the sources of information available on internet [39], whereas considering the nature of medical science and its constant development, the choice of resources and up-to-date information is necessary for mothers and to avoid confusion.

The confirmation of others was a factor that affected the trust among the participants. In the study by Kassim, [35] many people believed that looking for maternal health information in books was a good idea as suggested by their friends and relatives. Thus, selecting a book was generally not a completely personal decision, and confirmation of others was effective in using this resource The results suggested the need for mothers to be acquainted with books by healthcare professionals, particularly midwives, as the first line of pregnancy services. The provision and distribution of appropriate books should also be made by the government and mass media.

In the present study, some participants emphasized that if the contents are familiar and they feel they have heard them before, they trust them more. In the study of Peterson-Clark [37], the participants said that if a website confirms what they already knew, they would be more likely to trust the website. Among other important indicators that participants have pointed out was the acceptance of information based on reason and logic, the number of referrers to a source, and etc. One of the most important indexes that some participants referred to was the rationality of the material. It might be said that the mother who has reached critical levels of health literacy use this index, because searching enables the person to think and retrieve information accurately.

The full text of health content whether it is complete or incomplete and the ability to understand and read information in various sources, especially the internet was another feature of health information evaluation that participants mentioned in this study. It should be noted that a woman’s ability to meet her information needs is influenced by her access to different sources of information and her ability to understand the information [40], because access to a large amount of information does not necessarily mean understanding that information [41]. Findings from several studies also indicated that it is very difficult to read a large percentage of web pages containing nutritional information during pregnancy [42, 43]. Therefore, it is necessary to improve the quality of information from different sources for their further use and proper planning.

In this study, some people had no specific criteria for assessing the validity of information. It might be said that these mothers were not paying any attention to the credibility assessment of information and needed some training in that regard.

The participants used to search several sources, and experts’ opinion and approval to find the most reliable information. Schrah et al. [44] stated that people tend to accept the recommendation from a source with the highest credibility, while evaluating and judging several sources and making the decision to choose the best one. However, the evaluation of resources is done by various ways, such as previous experience of the individual with the source, introduction by others and the reputation of a source among the people [45]. The results of a study by Sillence et al. [17]. indicated that most people merge online information sources with the advice of doctors, friends, and family members to guide behavior and make decisions. In pregnancy, due to the importance of maternal decisions about maternal and fetal health and the fear of mothers, seeking information from different sources is not unexpected. Perhaps, the participants in this study used a multi-source search approach and endorsement by a physician to ensure information and to use it.

It is worth noting that appropriate care is not only limited to the provision of information, but also the sharing of information [46]. In this study, most women talked to a healthcare professional about the information they received. However, some women searched information separately without consulting with a healthcare professional. Most pregnant women shared information from websites with their doctor [6, 47]. However, other studies have reported that a small percentage of internet users consult their physician about the health information they receive [9, 12]. It is therefore important for healthcare professionals to consider the need to discuss information that mothers may have obtained from other sources and help supplement or correct that information.

Another method of evaluation used by the participants was comparing the information obtained from different sources. In a study by Metzger [48], most participants preferred to compare the information received with various sources.

One of the important issues raised by the participants in creating a safe margin in the information obtained, especially to avoid confusion about the information obtained, was to identify and select a reliable source to receive health information. Shieh et al. [49] stated that unfamiliarity with the internet, search methods and lack of knowledge about authentic internet sources are serious factors influencing the information search behavior and access to reliable information. Lewallen and Côté-Arsenault [50] reported that although women search for information on the internet, they may not be skilled at finding the information they need. An Australian study that asked women about keywords used to search for information found that, keywords were chosen in such a way that women could not find the information they were looking for [51]. Therefore, it is necessary to consider appropriate methods and strategies for searching and familiarizing pregnant women with appropriate and reliable sources, which are not only specific to internet resources, but can also be used for resources such as books, magazines, files, educational programs and TV networks.

Another used by the participants to assess the validity of information was ignoring small contradictions in the information presented by different sources. Given the lack of study in this regard, the researcher believed that the importance of an issue is affected by the partial or incomplete contradiction, so that in critical issues even a very minor contradiction encourages individuals to seek further information to gain more credible information.

### Implications for policy and practice

According to the results, it is suggested that health policy makers plan to reduce the confusion of mothers about information by educating healthcare professionals, in particular midwives, to keep their information up-to-date, be aware of mother’s information-finding behaviors, know about resources with high credibility (including websites) in the field of pregnancy and encourage mothers to discuss their experiences of finding information. In the meantime, designing websites with valid content and taking into account the information needs of mothers during pregnancy, are among important measures that should be considered by health authorities. This is because internet is one of the most accessible sources of information and the majority of mothers at any time, especially when dealing with pregnancy problems, first refer to this source. Thus, it is important for mothers to know the reputable websites and get the answers to their questions from them. All these measures help mothers to resolve contradictions and reduce frequent visits to health centers because of lack of knowledge or wrong information.

### Study strengths and limitations

This qualitative study was conducted for the first time to explore and describe the experiences of pregnant women regarding the validation and evaluation of maternal health information in Tehran city, Iran. Despite the small sample size, strategies were used to enhance the validity and overall reliability of the data, including researcher triangulation, peers’ review, and the use of multiple coders to review and interpret the data. Limitations of this study included the fact that although the findings of a qualitative approach lead to a better understanding and insight of a subject, they cannot necessarily be generalized to a larger population with the same degree of certainty. However, the results can help us plan for the future.

## Conclusion

The results showed that mothers use their understanding and experience to make sure of their information and to avoid confusion, and they have different views on the verification and assessment of maternal health information during pregnancy based on their experiences. They use different sources that are logical and reasonable, but sometimes inaccurate, to ensure the accuracy of information.

## Data Availability

The datasets used and/or analyzed during the current study are available from the corresponding author on reasonable request.
